# The histone deacetylase inhibitor PCI-24781 impairs calcium influx and inhibits proliferation and metastasis in breast cancer

**DOI:** 10.7150/thno.48314

**Published:** 2021-01-01

**Authors:** Tianshu Yang, Pei Wang, Xin Yin, Jingyao Zhang, Miaomiao Huo, Jie Gao, Gen Li, Xu Teng, Hefen Yu, Wei Huang, Yan Wang

**Affiliations:** 1 Beijing Key Laboratory of Cancer Invasion and Metastasis Research, Department of Biochemistry and Molecular Biology, School of Basic Medical Sciences, Capital Medical University, Beijing, 100069, China.; 2 State Key Laboratory of Molecular Oncology, National Cancer Center/National Clinical Research Center for Cancer/Cancer Hospital, Chinese Academy of Medical Sciences and Peking Union Medical College, 100021 Beijing, China.; 3 Department of Clinical Laboratory, The Second Hospital of Shandong University, Jinan, 250033, Shandong province, China.

**Keywords:** PCI-24781, epidrug, anti-tumor, calcium signaling, RGS2

## Abstract

Histone deacetylases (HDACs) are involved in key cellular processes and have been implicated in cancer. As such, compounds that target HDACs or drugs that target epigenetic markers may be potential candidates for cancer therapy. This study was therefore aimed to identify a potential epidrug with low toxicity and high efficiency as anti-tumor agents.

**Methods**: We first screened an epigenetic small molecule inhibitor library to screen for an epidrug for breast cancer. The candidate was identified as PCI-24781 and was characterized for half maximal inhibitory concentration (IC_50_), for specificity to breast cancer cells, and for effects on carcinogenesis and metastatic properties of breast cancer cell lines *in vitro*. A series of in silico and *in vitro* analyses were further performed of PCI-24781 to identify and understand its target.

**Results**: Screening of an epigenetic inhibitor library in MDA-MB-231 cells, a malignant cancer cell line, showed that PCI-24781 is a potential anti-tumor drug specific to breast cancer. Ca^2+^ related pathways were identified as a potential target of PCI-24781. Further analyses showed that PCI-24781 inhibited Gαq-PLCβ3-mediated calcium signaling by activating the expression of regulator of G-protein signaling 2 (RGS2) to reduce cell proliferation, metastasis, and differentiation, resulting in cell death in breast cancer. In addition, RGS2 depletion reversed anti-tumor effect and inhibition of calcium influx induced by PCI-24781 treatment in breast cancer cells.

**Conclusions**: We have demonstrated that PCI-24781 is an effective anti-tumor therapeutic agent that targets calcium signaling by activating RGS2. This study also provides a novel perspective into the use of HDAC inhibitors for cancer therapy.

## Introduction

Histone deacetylases (HDACs) regulate a series of key biological processes, such as cell cycle, cell death, DNA damage repair, and chromatin dynamics, by suppressing histone acetylation. In humans, the HDAC family consists of 18 genes, which can be grouped into four classes (class I-IV) on the basis of homology. HDACs manipulate transcriptional activity and remodel the chromatin by controlling the interaction of positively charged histones with negatively charged DNA 1. Studies have revealed that HDACs are highly expressed in a variety of tumors and can play a critical role in cancer progression by reversing the transformed phenotype, inhibiting cell proliferation, triggering cell cycle arrest, and inducing apoptosis 2. HDAC inhibitors have also been reported to reduce tamoxifen resistance and effectively delay disease progression with marginal effects on non-tumor tissue 3, 4. Therefore, identifying HDAC inhibitors with anti-tumor effects could be beneficial to the selection of clinical anti-cancer drugs.

The pan-HDAC inhibitor PCI-24781 is an orally bioavailable compound based on phenyl hydroxamic acid. PCI-24781 is widely studied as an anti-tumor drug in several cancer types, including breast cancer, gallbladder cancer, and lymphoid malignancies 5. PCI-24781 binds to the cofactor Zn^2+^ required for the catalytic function of the classical HDACs and inhibits HDAC1, 2, 3, 6, 8, and 10. Thus, it is an effective inhibitor of HDAC I and IIb. PCI-24781 exhibits different inhibition constants (K_i_) for different HDACs, ranging from 70 nM (HDAC1) to 280 nM (HDAC8) 6. The anti-tumor mechanisms of PCI-24781 have been revealed in different cancers. In colon tumor cells, PCI-24781 targets the RAD51 recombinase, leading to the reduction of cellular ability for homologous recombination, which hampers the repair of DNA double-strand breaks, leading to impaired genomic integrity and enhanced radiation sensitivity of tumors 5. In breast cancer, low doses of PCI-24781 have been observed to regulate cancer stem cell differentiation, which led to inhibited cell proliferation and migration; this was observed to be accompanied by low expression levels of a long non-coding RNA *Xist*, which can be used as a biomarker for breast cancer cell response to PCI-24781 7. Furthermore, HDAC inhibitors have been reported to enhance the epigenetic modifications in estrogen receptor (ER)-positive breast cancer cells, thereby affecting cancer progression, leading to cell death in breast cancer 8. However, the antitumor mechanisms of PCI-24781 in breast cancer are still limited. Further research on PCI-24781 is needed to elucidate the mechanisms underlying the effects of PCI-24781 in breast cancer.

As a second intracellular messenger, Ca^2+^ triggers and participates in a variety of cellular processes, including cell proliferation, migration, and survival. Calcium signaling is a potent and multi-faceted tool in achieving multiple cell outcomes. The regulation of various cellular mechanisms in cancer progression, such as cell proliferation, invasion, and death, is partially driven by calcium signaling 9, 10. In addition, calcium signaling plays crucial roles in maintaining and affecting drug resistance and in regulating the tumor microenvironment 11. Dysregulation of Ca^2+^ homeostasis may contribute to tumor cell death. For example, in breast cancer, overexpression of plasmalemmal Ca^2+^ pump isoform 2 increased resistance to apoptosis by controlling the magnitude and frequency of the intracellular calcium 12. In addition, the disruption of Ca^2+^ transport from the endoplasmic reticulum to the mitochondria led to cell death in some breast cancer cell lines. Studies have found that Ca^2+^-related proteins are aberrantly expressed in some cancers, and specific tumor suppressor mechanisms exhibited sensitivity to certain calcium signals 13. Intracellular Ca^2+^ can be regulated by key proteins such as the matrix metalloproteinases (MMP) and phospholipase C-β (PLCβ). The MMP family consists of 26 members that can regulate intracellular Ca^2+^ by degradation of the extracellular matrix (ECM) proteins 14. The four isoforms (β1-β4) of PLCβ produce diacylglycerol and inositol-1, 4, 5-triphosphate (IP_3_) to mobilize Ca^2+^, enhancing cell proliferation 15.

In this study, we explored the anti-tumor mechanisms of an epidrug, a pan-HDAC inhibitor PCI-24781, which has been reported to have a remarkable ability to suppress cell survival in breast cancer. We aimed to screen an epidrug with low toxicity and high efficiency as anti-tumor agent and explored the molecular mechanism of the candidate. These results bring a novel perspective for epidrug therapy, especially for breast cancer treatment.

## Materials and methods

### Cell culture and treatment

HeLa, MCF-7 and MDA-MB-231 cell lines were purchased from the American Type Culture Collection. MCF-10A, T-47D, PANC-1, A549, HCT-15, Caco-2, SMMC-7721, SH-SY5Y, HUVEC and PC-3 cell lines were obtained from the Chinese Academy of Medical Sciences. MEF was isolated from pregnant mice at 14‒16 days of pregnancy. Primary breast cancer cells were isolated from breast cancer patients. All media including Dulbecco Modified Eagle Medium (DMEM), RPMI 1640 Medium and DMEM/Nutrient Mixture F-12 were supplemented with 10% fetal bovine serum (FBS), 100 units/mL penicillin, and 100 mg/mL streptomycin (Gibco, Waltham, MA, USA). Cells were cultured in a humidified incubator equilibrated with 5% CO_2_ at 37 °C. Cells were treated for 24-72 h in adherent conditions with compounds from an epigenetic small molecule inhibitor library (L1200, Topscience, Shanghai, China).

### Cell Counting Kit-8 (CCK8) assay

The IC_50_ of PCI-24781 were evaluated through the CCK8 assay. HeLa, MCF-7, MDA-MB-231, T-47D, PANC-1, A549, HCT-15, Caco-2, SMMC-7721, SH-SY5Y, and PC-3 were seeded in 96-well plates at a density of 5 × 10^3^ cultured with 5% CO_2_ at 37 °C for 24 h and incubated with PCI-24781 for 72 h by gradient concentration (0.1 nM-10 nM) in culture medium. Cell viability was then detected using a CCK8 assay (MedChemExpress, USA) according to the manufacturer's instructions.

### Western blot

Total protein was assessed by sodium dodecyl sulfate-polyacrylamide gel electrophoresis using 10% or 15% gels, followed by immunoblotting with the following antibodies. Anti-glyceraldehyde 3-phosphate dehydrogenase (GAPDH) antibody (AC002) was purchased from ABclonal Technology (Woburn, MA, USA); anti-PLCβ3 antibody (14247) was purchased from Cell Signaling Technology (Danvers, MA, USA); anti-β-actin antibody (ab8226) and anti-histone H3 antibody (ab1791) were purchased from Abcam (Cambridge, UK); anti-ace tubulin antibody (66200-1-lg) was purchased from Proteintech Group (Rosemont, IL, USA); anti-γ-catenin antibody was purchased from BD Biosciences (Franklin Lakes, NJ, USA). The GAPDH and β-actin content were analyzed as the loading control.

### EdU incorporation assay

MDA-MB-231 cells were treated with 100 nM or 200 nM PCI-24781 for 24 h or 48 h, and then trypsinized and seeded into 6-well plates at a density of 0.8 × 10^5^ cells/ml to adhere overnight. After that, DNA proliferation was detected using an EdU assay kit according to the manufacturer's instructions (RiboBio, Guangzhou, China).

### TUNEL assay

Apoptosis was detected using a Cell Death Detection Fluorescein Kit for TUNEL assay (RiboBio, Guangzhou, China) according to the manufacturer's instructions. MDA-MB-231 cells were treated with either 100 nM or 200 nM PCI-24781 for 24 h or 72 h, then digested and seeded into 6-well plates at a density of 0.8 × 10^5^ to adhere overnight. Signals were detected by fluorescence microscopy. Non-apoptotic cells were visualized under a DAPI (4′,6-diamidino-2-phenylindole) filter.

### Colony formation assay

The colony-formation ability of MDA-MB-231 and T-47D cells were measured by the clonogenic assay. MDA-MB-231 and T-47D were seeded into 10 cm plates and treated with PCI-24781. After treatment, cells were digested and suspended in culture medium at a density of 5 × 10^3^ cells/ml. Cells (2,000 cells per well) were seeded into 6-well plates and cultured for two weeks. The, cells were fixed with 4% formaldehyde and washed with phosphate-buffered saline (PBS) and stained with crystal violet for half an hour. Images of colonies were captured using a light microscope.

### Wound-healing assay

Tumor cell migration was determined by the wound healing assay. MDA-MB-231 cells were seeded into 6-well plates and treated with 100 nM or 200 nM PCI-24781 for 24, 48, or 72 h. T-47D cells were seeded into 6-well plates and treated with 2 μM or 2.5 μM PCI-24781 for 24 or 48 h. After treatment, a straight scratch was made onto the cell monolayer using a pipette tip. The cells were washed with PBS three times to remove the floating cells, and the cells were cultured in serum-free medium for 24 h. Cell wound edges were captured using a light microscope.

### Cell invasion assay

Transwell chamber filters (BD) were coated with Matrigel diluted (1:10) in serum-free medium. MDA-MB-231 cells were seeded into 10 cm plates and treated with 100 nM or 200 nM PCI-24781 for 24, 48, or 72 h. T-47D cells were seeded into 10-cm plates and treated with 2 μM or 2.5 μM PCI-24781 for 24 or 48 h. After treatment, cells were digested and suspended in serum-free media. Cells (8,000 cells per well) were seeded into the upper chamber of the transwell chambers, and the chambers were transferred into 24-well plates containing 500 μl culture medium per well. After 20 h of incubation, cells in the upper chamber were fixed with 4% formaldehyde, washed with PBS, and stained with crystal violet for half an hour. Images of invasive cells were captured using a light microscope.

### RNA-sequencing analysis

MDA-MB-231 cells treated with PCI-24781 or DMSO for 24 h or 48 h. Total RNA was extracted using a TRIzol reagent (Roche, Basel, Switzerland) according to the manufacturer's instructions. The product was sent to the Beijing Genomics Institute (BGI, Shenzhen, China) for mRNA library construction and sequencing including mRNA enrichment or rRNA depletion, second strand synthesis and bubble adapter ligation. The sequencing data verified by a quality control step were used for further analysis. The listed results of the RNA-seq analysis are summarized in **Table S1**. All RNA-seq data are available on https://www.ncbi.nlm.nih.gov/geo/query/acc.cgi?acc=GSE150038.

### Real-time quantitative PCR analysis

For quantification of mRNA of target genes by RT-qPCR, total RNA was extracted from MDA-MB-231 cells treated with PCI-24781 using a TRIzol reagent (Roche, Basel, Switzerland). Reverse transcription was performed using the RevertAid First Strand cDNA Synthesis Kit (Roche, Basel, Switzerland) according to the manufacturer's instructions. The primers for the detection of target mRNA are shown in **Table S2**. β-actin was used as an internal reference. The relative expression of target genes was calculated by the 2^-ΔΔCt^ method.

### Intracellular calcium measurement

MDA-MB-231 cells were seeded into 10-cm plates and treated with 100 nM or 200 nM PCI-24781 for 24, 48, or 72 h. After treatment, the cells were digested and seeded in 96-well black plates (Corning Costar, Cambridge, MA, USA) and cultured for 20 h. For measurement of [Ca^2+^]_i_, the cells were labeled with Fluo-4-AM in serum-free medium for half an hour at room temperature. After washing with Dulbecco's Phosphate Buffered Saline (D-PBS), cells were overlaid in Hank's balanced salt solution (5.4 mM KCl, 1 mM MgCl_2_, 20 mM hydroxyethyl piperazineethanesulfonic acid (HEPES), 120 mM NaCl, 10 mM glucose, 0.2 mM Ethylenebis(oxyethylenenitrilo)tetraacetic acid (EGTA)). Continuous fluorescence intensity was detected after thrombin was added as a calcium agonist by an automated sampler. Response over baseline was assessed as a relative measure of [Ca^2+^]_i_.

### Chromatin immunoprecipitation (ChIP)

ChIP was performed with PCI-24781-treated and untreated MDA-MB-231 cells. Briefly, the cells were cross-linked with 1% formaldehyde, sonicated, pre-cleared, and incubated with 4 μg of Ace-H3 antibody or IgG, which was followed by the addition of Dynabeads Protein G. Next, the beads were washed with high- and low-salt-concentration buffers, and DNA was eluted and purified using the QIAquick PCR Purification Kit. The amount of the DNA template was analyzed by qPCR using primers specific for five promoter regions. The primer pairs used are listed in**Table S3**.

### Animal models

MDA-MB-231 (5 × 10^6^ cells) were inoculated into the left abdominal mammary fat pad of 6-week-old female nude mice, and tumor growth was monitored. Fourteen mice were divided into two groups: control and PCI-24781-treated group. When the tumor size was approximately 100 mm^3^, we initiated treatment with PCI-24781 dissolved in 50 mM sodium lactate buffer (pH 4.2) (i.v., 50 mg/kg, every other day for four weeks). After four weeks of treatment, all mice were sacrificed according to ethical guidelines. Tumors were excised and analyzed. Tumor volume was measured using a Vernier caliper and calculated according to the following formula: π/6 × length × width^2^. All animal handling and procedures were approved by the Capital Medical University Institutional Animal Care.

### Immunochemistry staining

Tumor and adjacent tissues were rapidly fixed in 4% paraformaldehyde (Sigma-Aldrich) for at least 24 h and embedded in paraffin for microscopic examination. From the prepared paraffin blocks, 5-μm thick sections were cut and stained with hematoxylin and eosin (H&E) for light microscopic observation. Sections were processed as per standard protocols using 3,3'-diaminobenzidine (DAB) staining. All human and mouse tissues were collected according to protocols approved by the Ethics Committee of Capital Medical University, and informed consent was obtained from all patients.

### Statistical analysis

Statistical analyses of all data were performed using GraphPad Prism (version 8.0, Graph Pad Software Inc., San Diego, CA, USA). All the data were analyzed by Student's *t*-test or one-way ANOVA. The Z-Score was chosen to evaluate the results and experimental deviation. Quantitative data are presented as the mean ± SD. The asterisk (*) denotes statistically significant difference (*P* < 0.05). IC_50_ was calculated using a four-parameter fit with variable slope in GraphPad Prism.

## Results

### High-content epigenetic inhibitor screen identifies PCI-24781 in breast cancer

**Figure 1:**
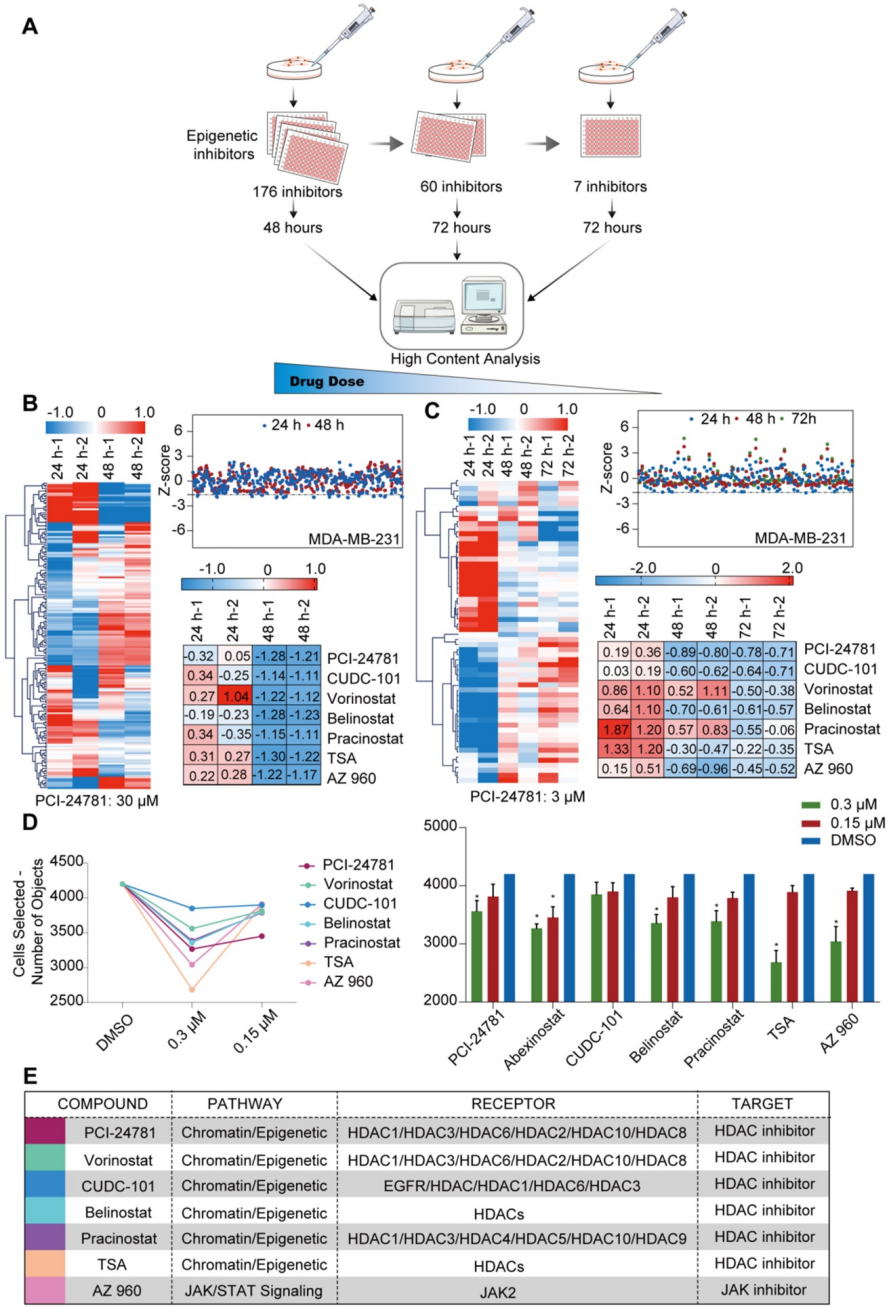
** A high-content epigenetic inhibitor screen identifies PCI-24781 in breast cancer. A.** The selection strategy for screening multiple compounds at different concentrations of the epigenetic small molecule inhibitor library. **B.** Quantification of living cells after treatment with 167 epigenetic compounds (30 µM each) for 24 and 48 h. The Z-Score was chosen to evaluate the results and experimental deviation (n = 3). TSA, Trichostatin A. **C.** Quantification of living cells after treatment with 60 epigenetic compounds (3 µM each) for 24, 48, and 72 h. The Z-Score was chosen to evaluate the results and experimental deviation (n = 3). **D.** Quantification of living cells after treatment with 7 epigenetic compounds (0.3 µM and 0.15 µM) for 72 h. The Z-Score was chosen to evaluate the results and experimental deviation (n = 3; *P < 0.05). **E.** Identities of the 7 compounds that were identified in the screen, including pathway, receptor, and target of each compound.

To identify potential anti-tumor inhibitors, we screened an epigenetic small molecule inhibitor library for anti-tumor activity in breast cancer cells. One aim of our research was to develop an effective selection strategy for screening multiple compounds at different concentrations. The selection strategy mainly comprised three stages, in which different concentrations (high to low) of inhibitors screened with a high-content analysis instrument were identified using cancer cell lines in a 96-well plate format (**Figure 1A**). For the first step, we treated cells with a high concentration of compounds to exclude compounds ineffective in killing cancer cells. MDA-MB-231 cells, a human breast cancer cell line, were treated with 167 epigenetic compounds (30 μM each) for 48 h, and then analyzed by high-content analysis, where changes in cell morphology relative to the control (dimethyl sulfoxide, DMSO) would indicate modulation of cancer cells by compound treatment. Of the tested compounds, 107 were ruled out, and 60 compounds were selected for the next step. These included HDAC inhibitors, Janus kinase (JAK) inhibitors, mammalian target of rapamycin (mTOR) inhibitors, a histone methyltransferase inhibitor, an Aurora kinase inhibitor, a hypoxia-inducible factor (HIF) inhibitor, and a protease inhibitor (**Figure 1B**). At the second step, we treated the cells with the 60 compounds at a median concentration (3 μM) for 72 h to screen for inhibitors that can kill cancer cells with high efficiency. HDAC inhibitors and JAK inhibitors, including PCI-24781, vorinostat, CUDC-101, belinostat, pracinostat, trichostatin A (TSA), and AZ960, had discernible effects on cell survival (**Figure 1C, E**). At the third step, breast cancer cells were exposed for 72 h to decreasing concentrations (0.3 μM and 0.15 μM) of the 7 inhibitors to explore the most effective inhibitor at a low dose. Based on the high-content analysis, PCI-24781, TSA, and AZ960 treatment resulted in 20%‒40% cell death at 0.3 μM. However, the HDAC inhibitor PCI-24781 was the only compound that displayed ~20% cell death at the lowest concentration (0.15 μM) (**Figure 1D**). To predict the absorption, distribution, metabolism, and excretion (ADME) of PCI-24781, we used the online tool ADMETlab (http://admet.scbdd.com/) to predict the ADME of PCI-24781 (**Table 1**). The prediction of ADME properties revealed that PCI-24781 exhibits high bioavailability, low drug interaction potential, and relative low side effects, indicating the potential of PCI-24781 as an orally active inhibitor. Taken together, based on the results of screening a small molecule epigenetic inhibitor library in MDA-MB-231, we found that PCI-24781 was the most effective inhibitor of breast cancer cells. Our results suggest that PCI-24781 is a promising therapeutic agent against breast cancer.

### PCI-24781 is selective to breast cancer cells

**Figure 2:**
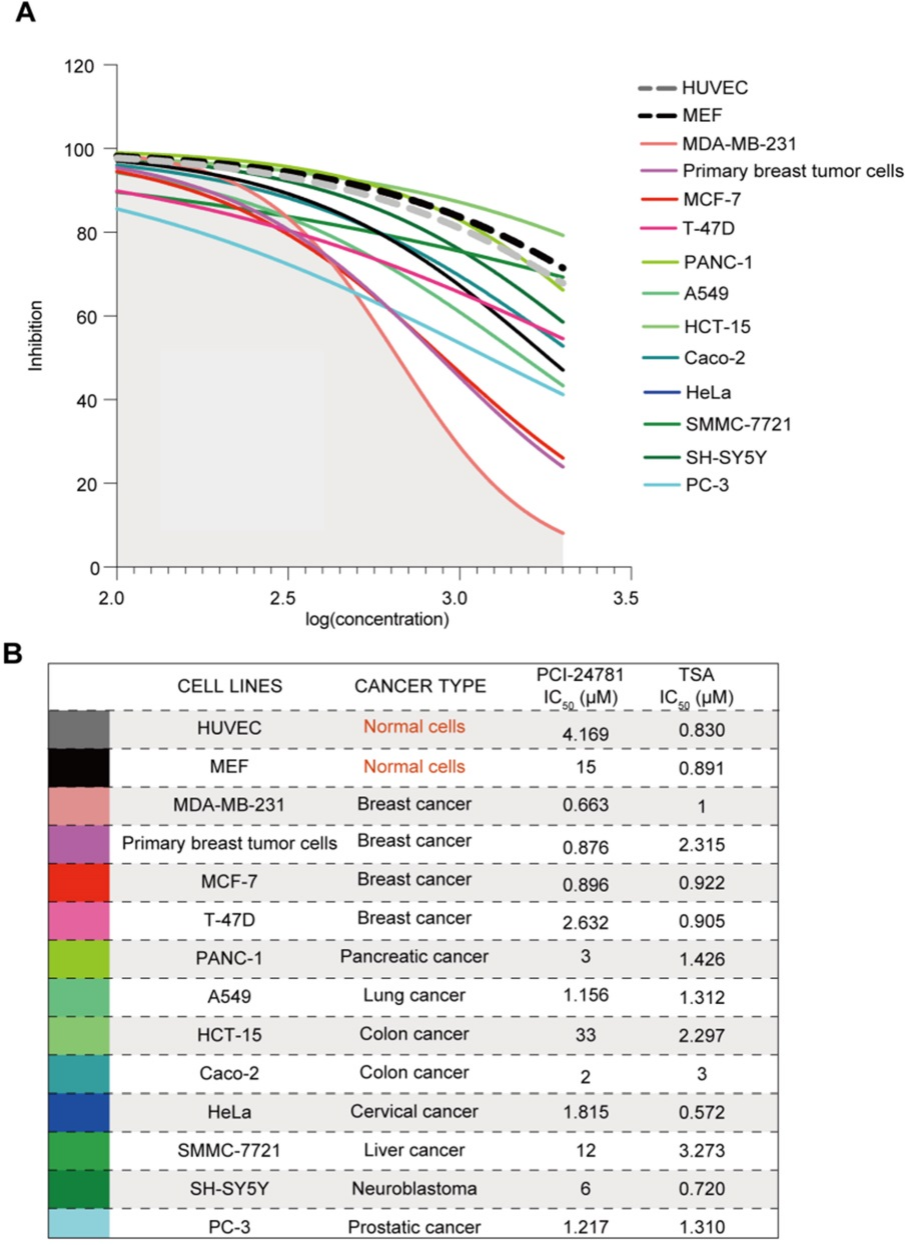
** PCI-24781 is selective to breast cancer cells. A-B.** Measurement of IC_50_ by CCK8 assay in cell panels (including MEF, HUVEC, primary breast tumor cells, MDA-MB-231, MCF-7, T-47D, PANC-1, A549, HCT-15, Caco-2, HeLa, SMMC-7721, SH-SY5Y, and PC-3) treated with PCI-24781 and TSA (n = 3).

In order to determine if PCI-24781 is a breast cancer-specific drug or a broad-spectrum anti-tumor drug, we measured the half maximal inhibitory concentrations (IC_50_) of PCI-24781 and the HDAC inhibitor TSA in several tumor cell lines, including primary breast cancer cells. Cell lines for breast, pancreatic, lung, colon, cervical, liver, prostatic cancer and neuroblastoma were exposed for 72 h to increasing concentrations (100 nM-2 μM) of PCI-24781 and TSA (**Figure S1A**). We also chose to use mouse embryonic fibroblasts (MEF) and Human Umbilical Vein Endothelial Cells (HUVEC) to evaluate the effects of PCI-24781 on normal cells. As shown in **Figure S1A**, normal cells exhibited strong sensitivity to TSA treatment, as evidenced by low IC_50_ values of <1 μM. More importantly, TSA acted as a broad-spectrum anti-tumor drug, exhibiting uniform anti-tumor activity in all tumor cell lines, with no preference for a certain tumor type. In contrast, MEF and HUVEC were not sensitive to PCI-24781 (IC_50_ = 15 μM and 4.2 μM, respectively); however, all but the non-breast cancer cell lines were classified as non-sensitive to low doses of PCI-24781, with IC_50_ > 1 μM (IC_50_ range: 1.217‒33 μM). MDA-MB-231 and MCF-7 cells were classified as sensitive to low concentrations of PCI-24781 with respective IC_50_ values of 663 and 896 nM (**Figure 2A-B**). These results indicate that the target cells of PCI-24781 treatment are breast cancer cells, revealing cell specificity.

### PCI-24781 treatment inhibits breast carcinogenesis and metastasis

**Figure 3:**
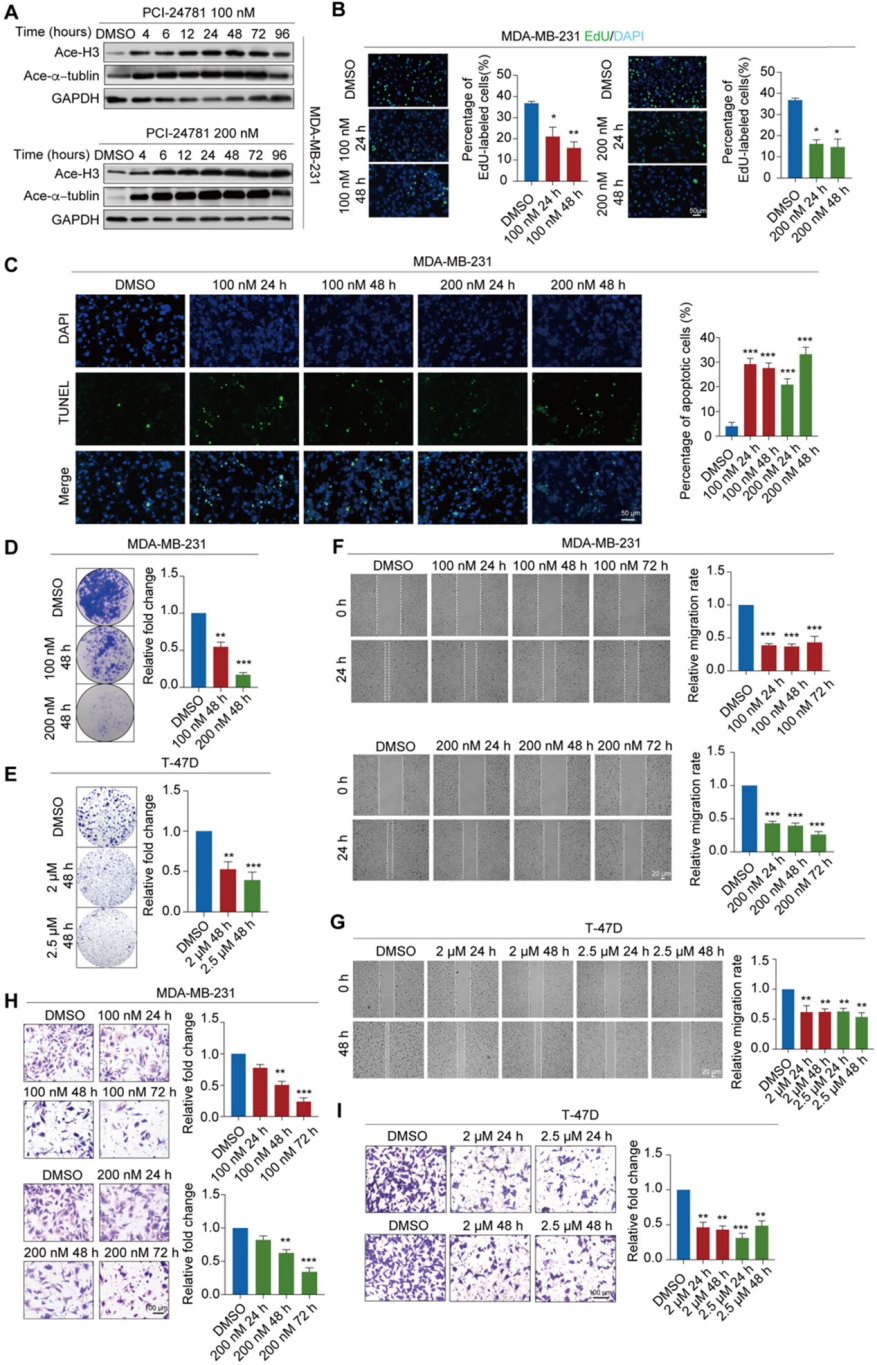
** PCI-24781 treatment inhibits breast carcinogenesis and metastasis. A.** Western blot using the indicated antibodies were performed on total protein extracted from MDA-MB-231 cells treated with PCI-24781. Ace-H3, acetylated histone H3; Ace-α-tublin, acetylated α-tublin. **B.** EdU incorporation assays were performed on MDA-MB-231 cells treated with PCI-24781. Representative images are shown on the left, and statistical analysis is shown on the right (*P < 0.05, **P < 0.01). Scale bars, 50 µm. **C.** TUNEL assays were performed on MDA-MB-231 cells treated with PCI-24781 treatment. Representative images are shown on the left, and statistical analysis is shown on the right (***P < 0.001). Scale bars, 50 µm. **D and E.** MDA-MB-231 and T-47D cells treated with PCI-24781 were cultured for 10 days prior to crystal violet staining. Representative images are shown on the left, and statistical analysis is shown on the right (**P < 0.01, ***P < 0.001). **F and G.** Wound-healing assays were performed in MDA-MB-231 and T-47D cells treated with PCI-24781. Representative images are shown on the left, and statistical analysis is shown on the right (**P < 0.01, ***P < 0.001). Scale bars, 20 µm. **H and I.** Cell invasion assays were performed using the matrigel transwell filters in MDA-MB-231 and T-47D cells treated with PCI-24781. Invading cells were stained and counted. Representative images are shown on the left, and statistical analysis is shown on the right (**P < 0.01, ***P < 0.001). Scale bars, 100 µm.

To determine whether PCI-24781 has inhibitory effects on histone deacetylase, we measured acetylated histone and acetylated tubulin levels after PCI-24781 treatment. MDA-MB-231 cells were exposed to different concentrations (100 or 200 nM) of PCI-24781 for 24-72 h to investigate the optimal treatment conditions in this cell line (**Figure 3A**). Treating MDA-MB-231 cells with PCI-24781 resulted in time-dependent and dose-dependent accumulation of acetylated histone H3 (H3) and acetylated α-tubulin, revealing that histone deacetylase is suppressed. Optimal suppression of histone deacetylase was observed upon treatment with 100 nM or 200 nM PCI-24781 for 24 h.

We further verified the anti-tumor activity of PCI-24781 treatment in MDA-MB-231, T-47D, and ER-positive primary breast tumor cells by detecting apoptosis, cell proliferation, cell growth, cell invasion, and cell migration (**Figure S2A**). We found that, compared to DMSO treatment, PCI-24781 treatment led to decreased proliferation of MDA-MB-231 and primary breast tumor cells based on the 5-ethynyl-2'-deoxyuridine (EdU) incorporation assay (**Figure 3B, Figure S2C**). Moreover, based on the TUNEL assay, MDA-MB-231 cells treated with PCI-24781 had a higher rate of apoptosis than cells treated with DMSO (**Figure 3C**). *In vitro* colony formation assays and crystal violet assays showed decreased colony formation in MDA-MB-231, T-47D, and primary breast tumor cells treated with PCI-24781 (**Figure 3D-E, Figure S2D**). We detected PCI-24781-induced regulation of cellular migration of breast cancer cells by the wound healing assay and found that PCI-24781 treatment induced a significant delay (*P* < 0.05) in wound closure compared with DMSO treatment in MDA-MB-231, T-47D, and primary breast tumor cells (**Figure 3F-G, Figure S2E**). We next examined the invasive capacities of breast cancer cells by the transwell invasion assays and found that PCI-24781 treatment led to significant reduction in the invasive capacity of breast cancer cells (*P* < 0.05) (**Figure 3H-I, Figure S2F**). Altogether, these results reveal that PCI-24781 treatment inhibits both breast carcinogenesis and breast cancer metastasis.

### Genome-wide identification of transcription signaling for PCI-24781 treatment

**Figure 4:**
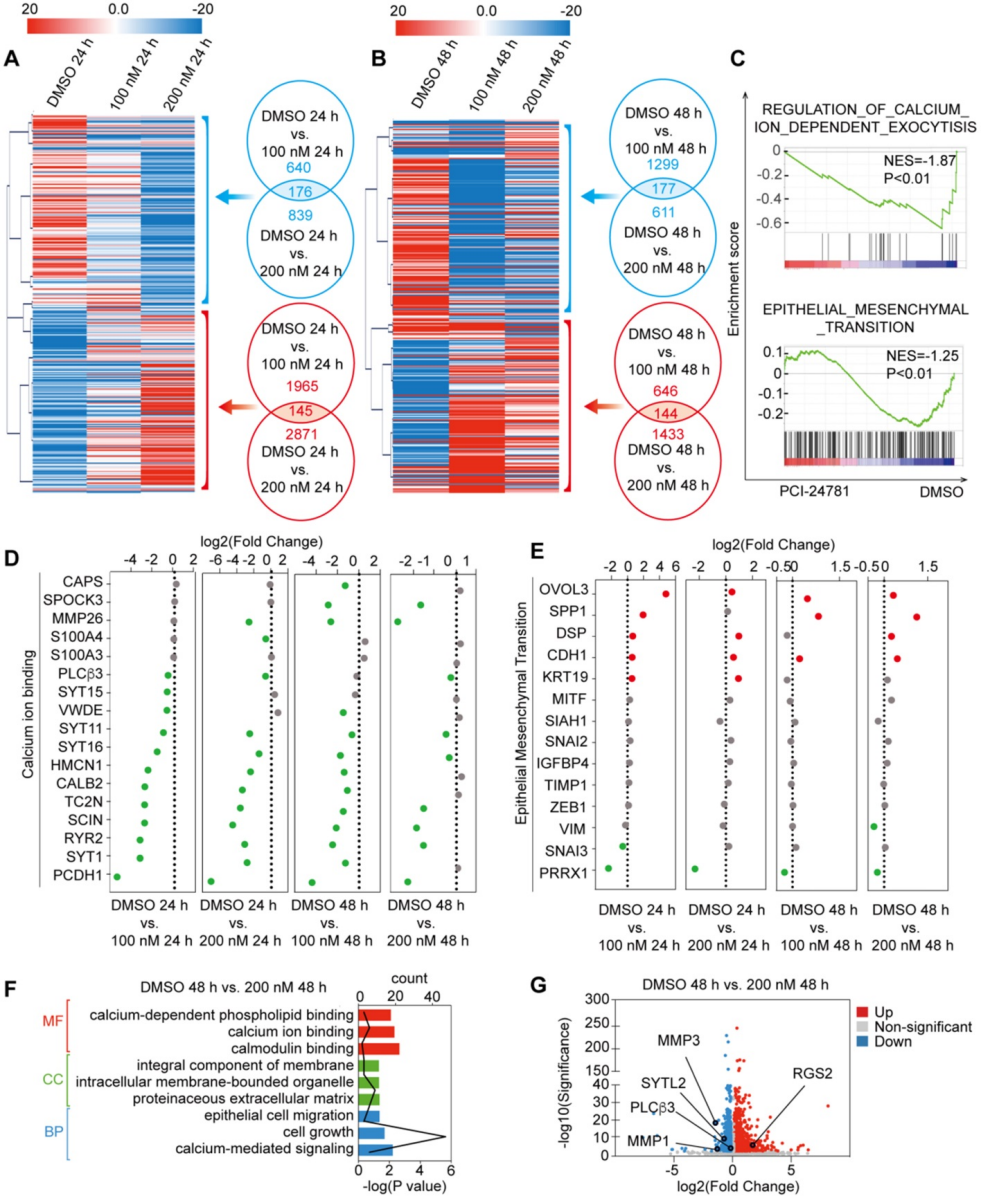
** RNA-seq analysis of MDA-MB-231 with PCI-24781 treatment. A and B.** Heat maps of the features of expression profile changes in MDA-MB-231 cells treated with PCI-24781. mRNA expression data were clustered using the MeV 4.8 software. **C.** GSEA analysis of the differences in gene expression after treatment with PCI-24781. **D and E.** Analysis of the expression of calcium-ion binding and EMT pathways in the RNA-seq analysis. **F.** GO enrichment analysis of the differences in gene expression after PCI-24781 treatment was performed to identify the targets of PCI-24781 in gene regulation. **G.** Volcano plot is shown to identify the differentially expressed genes.

To achieve a gene-by-gene understanding of the effectors regulated by PCI-24781, we assessed the effects of treatment with 100 nM and 200 nM PCI-24781 on gene expression in MDA-MB-231 cells through RNA sequencing (RNA-seq) analysis. Four independent sample groups were exposed to PCI-24781 and controls were treated with DMSO for 24 h and 48 h (DMSO-treated groups: DMSO 24 h, DMSO 48 h; PCI-24781-treated groups: 100 nM 24 h; 100 nM 48 h; 200 nM 24 h; 200 nM 48 h) and were analyzed through RNA-seq. Significant changes in the gene expression profile of MDA-MB-231 cells were observed after PCI-2478 treatment (**Figure 4A-B**). As shown in **Figure 4A**, the mRNA levels were clustered, and we observed hundreds of genes with significantly decreased or increased expression after PCI-24781 treatment. Four pairwise comparisons between the DMSO- and PCI-24781-treated groups (DMSO 24 h vs. 100 nM 24 h; DMSO 24 h vs. 200 nM 24 h; DMSO 48 h vs. 100 nM 48 h; and DMSO 48 h vs. 200 nM 48 h) are presented to show differential gene expression. Overlapping the upregulated or downregulated genes in each of the pairwise comparisons, we found 145 upregulated and 176 downregulated genes in the PCI-24781-treated group 24 h relative to the DMSO 24 h group, and we found 144 upregulated and 177 downregulated genes in the PCI-24781-treated 48 h group compared to the DMSO 48 h group. To explore the top gene signaling pathways that were augmented or abated by PCI-24781 treatment, we performed Gene Set Enrichment Analysis (GSEA), which identified significant inactivation of the Ca^2+^-related pathway and the epithelial-mesenchymal transition (EMT) pathway (**Figure 4C**). In order to further explore the aspects of related genes in the Ca^2+^-related pathway and the EMT pathway, we analyzed the expression of 20 genes in the Ca^2+^-binding pathway including *TC2N*, *SCIN*, *RYR2*, *SYT1*, and *PCDH1*, and 15 genes expression of EMT pathway including *OVOL3*, *SPP1*, *CDH1*, *SNAI1*, *SNAI2*, and *ZEB1* (**Figure 4D-E**). We found that in the Ca^2+^-binding-related gene panel, these gene expression were mainly downregulated or unchanged, which meant that the Ca^2+^ relative pathway was inhibited. In EMT pathway-related gene panel, the transcription levels of epithelial markers were mainly augmented; on the contrary, the mesenchymal-related gene levels were mainly unchanged or decreased, suggesting that PCI-24781 treatment reversed EMT. To investigate the function of PCI-24781 in transcription signaling regulation, we performed Gene Ontology (GO) enrichment and Kyoto Encyclopedia of Genes and Genomes (KEGG) pathway analysis. We statistically integrated the GO and KEGG pathway analyses into the pairwise comparisons to select the top 5 pathways. We then found that the Ca^2+^-related pathways, including Ca^2+^ binding, calcium-mediated signaling, and calcium-dependent phospholipid binding, were the targets of PCI-24781 treatment (**Figure S3A**).

In these four pairwise comparisons, we found that the DMSO 48 h vs. 200 nM 48 h group revealed the most comprehensive information about transcription signaling genes, oncogenes, and tumor suppressor genes. The results of the GO analysis in the PCI-24781 200 nM 48 h group compared with the DMSO 48 h group showed that PCI-24781 treatment affected several key pathways involved in cancer modulation and several vital cellular responses, such as cell proliferation and differentiation (**Figure 4F**). A volcano plot of over 2,000 genes was shown to identify genes that are different between the DMSO 48 h group and PCI-24781 200 nM 48 h group. It showed that a series of Ca^2+^ signals markers and neuroendocrine (NE) differentiation markers were activated or inactivated including *SYTL2*, *MMP1*, *MMP3*, *PLCβ3*, and *RGS2* (**Figure 4G**).

**Figure 5:**
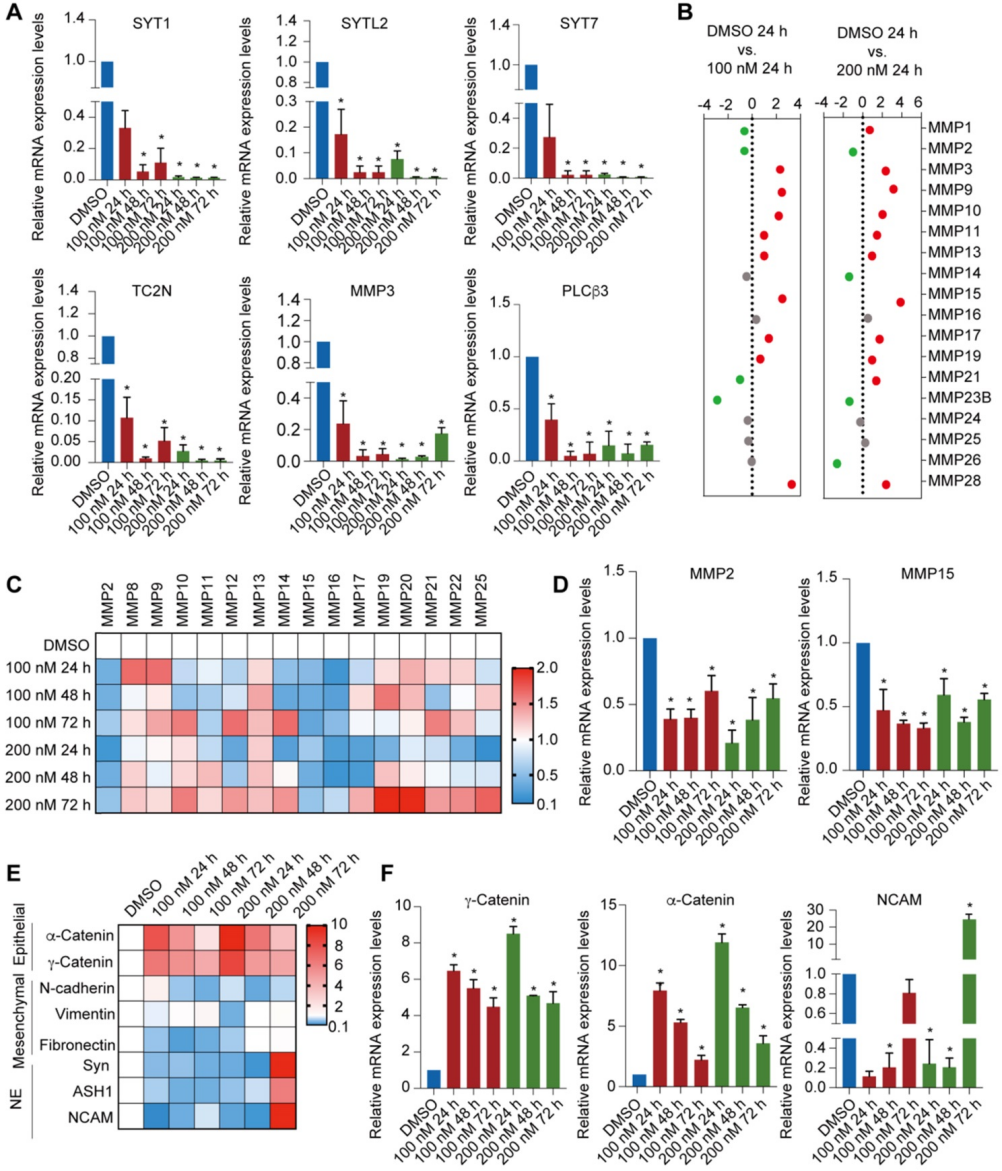
** Identification of PCI-24781 target genes on MDA-MB-231 cells by RNA-seq analysis. A.** RT-qPCR analyses for the expression of downregulated transcription targets of PCI-24781 in MDA-MB-231 cells. Bars represent the mean ± SD of triplicate cell cultures (*P < 0.05). **B.** Analysis of the expression of MMP families in RNA-seq analysis. **C and D.** RT-qPCR analyses of MMP family expression in MDA-MB-231 cells treated with PCI-24781. Bars represent the mean ± SD of triplicate cell cultures (*P < 0.05). **E and F.** RT-qPCR analyses of epithelial, mesenchymal, and neuroendocrine markers expression in MDA-MB-231 treated with PCI-24781. Bars represent the mean ± SD of triplicate cell cultures (*P < 0.05).

To validate the synergy pathways implicated by the RNA-seq analysis, we performed reverse transcription quantitative qPCR (RT-qPCR) for a subset of these marker genes. The expression of Ca^2+^-related pathway markers such as *SYT1*, *SYTL2*, *SYT7*, *SYT12*, *TC2N*, *MMP1*, *MMP3* and *PLCβ3* were downregulated after PCI-24781 treatment (**Figure 5A, Figure S3B**). To further explore the modulation of MMPs by PCI-34781, we assessed the MMP family gene panel in RNA-seq analysis and the mRNA levels of the MMP family members by RT-qPCR. Based on the RNA-seq analysis, expression of *MMP1*, *MMP2*, and *MMP3* mainly decreased after PCI-34781 treatment in all four pairwise comparisons (**Figure 5B, Figure S3C**). Although the results of RT-qPCR showed that the expression of *MMP2*, *MMP15*, and *MMP16* were significantly inhibited after PCI-24781 treatment, these results might involve the modulation of Ca^2+^-related pathways (**Figure 5C-D**). PCI-24781 treatment of MDA-MB-231 cells resulted in significant upregulation in the expression of epithelial markers such as α-catenin and γ-catenin, whereas the mRNA levels of mesenchymal/NE markers, including N-cadherin, vimentin, fibronectin, synaptophysin (Syn), absent small and homeotic disks protein 1 homolog (ASH1), and neural cell adhesion protein 1 (NCAM1) were either inhibited or unaffected (**Figure 5E-F**). These experiments support the hypothesis that PCI-24781 affects EMT progression and Ca^2+^ signaling pathways to suppress tumorigenesis.

### PCI-24781 treatment impedes calcium influx to inhibit cancer progression

**Figure 6:**
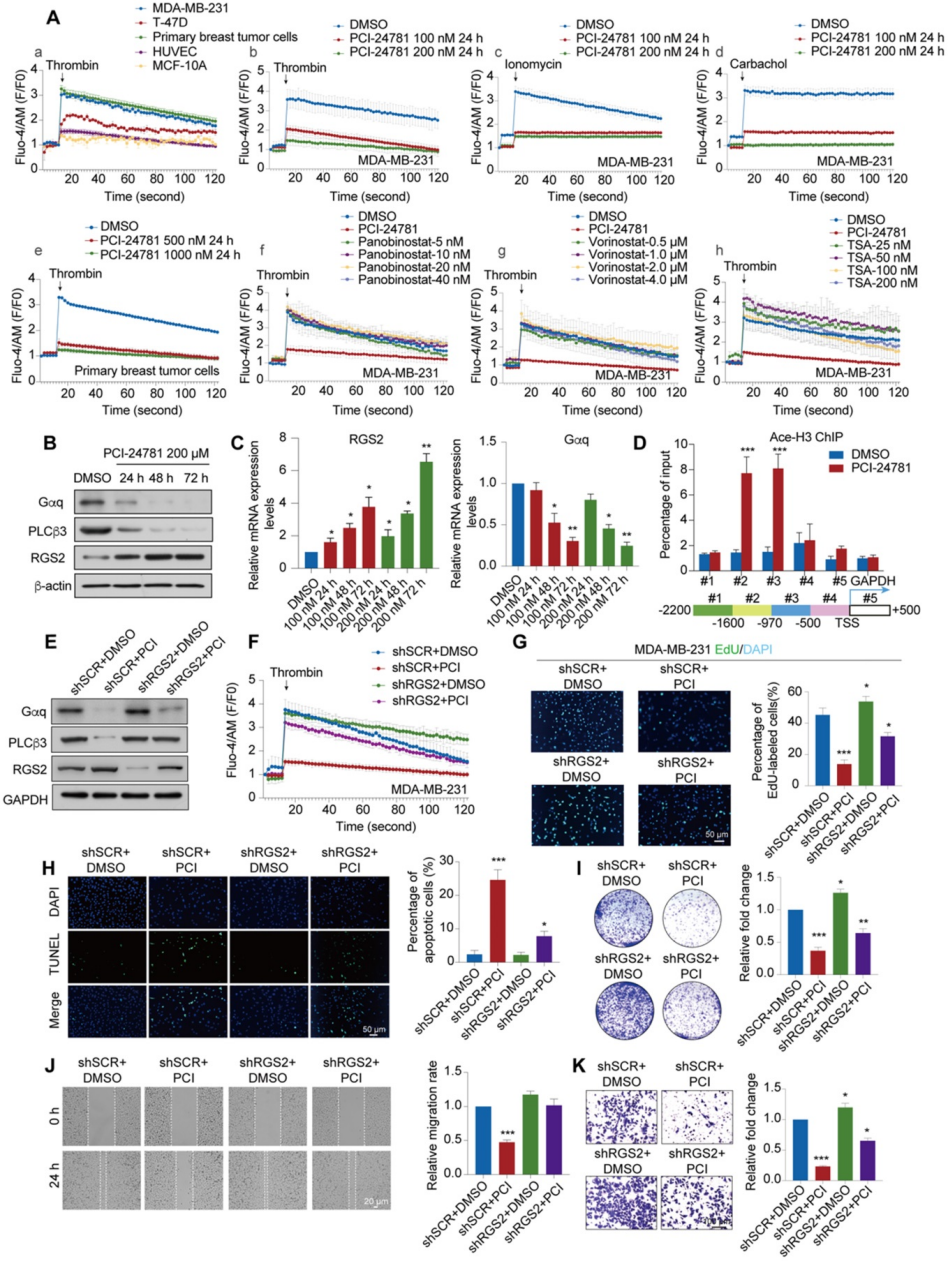
** PCI-24781 treatment impedes calcium influx to inhibit cancer progression via RGS2 activation. A.** The frequency and amplitude of [Ca^2+^]_i_ oscillations in response to thrombin stimulation in MDA-MB-231, T-47D, Primary breast tumor cells, HUVEC and MCF-10A (a). The frequency and amplitude of [Ca^2+^]_i_ oscillations in response to thrombin, ionomycin and carbachol stimulation in MDA-MB-231 with PCI-24781 treatment (b-d). The frequency and amplitude of [Ca^2+^]_i_ oscillations in response to thrombin stimulation in primary breast tumor cells with PCI-24781 treatment (e). The frequency and amplitude of [Ca^2+^]_i_ oscillations in response to thrombin stimulation in MDA-MB-231 with panobinostat, vorinostat and TSA treatment (f-h). n = 3, error bars represent the mean ± SD. **B.** Western blot with indicated antibodies of total protein extracted from MDA-MB-231 cells treated with PCI-24781 200 µM. **C.** RT-qPCR analyses of RGS2 and Gαq expression in MDA-MB-231 cells treated with PCI-24781. Bars represent the mean ± SD of triplicate cell cultures (*P < 0.05, **P < 0.01). **D.** qChIP-based promoter-walk assay in MDA-MB-231 cells after PCI-24781 treatment to map Ace-H3 enrichment in regions #2 and #3 of the RGS2 promoter. Error bars represent the mean ± SD of three independent experiments (***P < 0.001). **E.** Western blot analysis with indicated antibodies of total protein extracted from control, PCI-24781 treatment, RGS2 KD, and RGS2 KD with PCI-24781 treatment MDA-MB-231. shSCR, control scrambled shRNA. **F.** The frequency and amplitude of [Ca^2+^]_i_ oscillations in response to thrombin stimulation in control, PCI-24781 treatment, RGS2 KD, and RGS2 KD with PCI-24781 treatment MDA-MB-231; error bars represent the mean ± SD (n = 3). **G.** EdU incorporation assays were performed on control, PCI-24781 treatment, RGS2 KD, and RGS2 KD with PCI-24781 treatment MDA-MB-231. Representative images are shown on the left, and statistical analysis is shown on the right (*P < 0.05, ***P < 0.001). Scale bars, 50 µm. **H.** TUNEL assays were performed on control, PCI-24781 treatment, RGS2 KD, and RGS2 KD with PCI-24781 treatment MDA-MB-231. Representative images are shown on the left, and statistical analysis is shown on the right (*P < 0.05, ***P < 0.001). Scale bars, 50 µm. **I.** MDA-MB-231 shRGS2 cells treated with PCI-24781 were cultured for 10 days before crystal violet staining. Representative images are shown on the left, and statistical analysis is shown on the right (*P < 0.05, ***P < 0.001). **J.** Wound-healing assays were performed in control, PCI-24781 treatment, RGS2 KD, and RGS2 KD with PCI-24781 treatment MDA-MB-231. Representative images are shown on the left, and statistical analysis is shown on the right (***P < 0.001). Scale bars, 20 µm. **K.** Cell invasion assays were performed using the matrigel transwell filters in control, PCI-24781 treatment, RGS2 KD, and RGS2 KD with PCI-24781 treatment MDA-MB-231. Invading cells were stained and counted. Representative images are shown on the left, and statistical analysis is shown on the right (*P < 0.05, ***P < 0.001). Scale bars, 100 µm.

Given that PCI-24781 treatment may function through Ca^2+^-related pathways, including calcium ion binding and calcium-mediated signaling, which may involve calcium influx, we detected the changes in time-dependent intracellular calcium concentrations ([Ca^2+^]_i_) using the Ca^2+^ indicator Fluo-4-AM and the Ca^2+^ agonist thrombin *in vitro*. We first monitored the time-dependent changes in [Ca^2+^]_i_ in MDA-MB-231, MCF-7, and MCF-10A cells without PCI-24781 treatment to evaluate the levels of [Ca^2+^]_i_ in breast cancer cells with different molecular features as well as in mammary epithelial cells (**Figure 6A**). Interestingly, the amplitude of [Ca^2+^]_i_ oscillations in breast cancer (MDA-MB-231 and MCF-7) and primary breast cancer cells were higher than that in mammary epithelial cells (MCF-10A) and HUVEC, and the frequency of [Ca^2+^]_i_ oscillations in MDA-MB-231 was higher than that in T-47D, suggesting that the influx of intracellular Ca^2+^ is enriched in MDA-MB-231 (**Figure 6Aa**). As shown in **Figure 6Ab-c and Figure S3D**, we found that the influx of intracellular calcium ions induced by three different Ca^2+^ agonists thrombin, ionomycin, and carbachol treatment were completely inhibited in MDA-MB-231 cells after treatment with 100 nM PCI-24781 or 200 nM PCI-24781 for 24 h. The inhibitory effect of PCI-24781 on calcium influx was independent of the used Ca^2+^ agonist. In the primary breast cancer cells, thrombin treatment led to a significant decrease in the amplitude of [Ca^2+^]_i_ oscillations after PCI-24781 treatment (**Figure 6Ae**). To further explore whether other HDAC inhibitors affect calcium influx, we treated MDA-MB-231 cells with three pan-HDAC inhibitors (vorinostat, panobinostat, and TSA) and monitored the [Ca^2+^]_i_ oscillations (**Figure 6Af-h**). Analysis of [Ca^2+^]_i_ after treatment with different concentrations of the pan-HDAC inhibitors revealed that vorinostat, panobinostat, and TSA did not alter the frequency and amplitude of [Ca^2+^]_i_ oscillations, suggesting that the changes in the influx of intracellular calcium are specifically regulated by PCI-24781. These data suggest that PCI-24781 plays an important role in regulating calcium influx in breast cancer cells. As previously stated, studies have shown that the Ca^2+^ signals can modulate cancer progression by regulating migration, proliferation, cell stress, and apoptosis 11. Therefore, we conjecture that PCI-24781 treatment acts against cancer by inhibiting calcium influx.

The changes of Ca^2+^ signals are mainly related to the influx and efflux of calcium 16. The process of calcium influx can be determined by the promotion of the generation of inositol triphosphate (IP_3_) and release of Ca^2+^ from the ER via the activation of PLCβ3 by Gq-coupled GPCR signal. Besides, the process of calcium efflux can be driven by ATP-dependent plasma membrane calcium pump (PMCA). To explore the modulation of calcium influx and efflux by PCI-24781, we detected the expression of Gαq and PLCβ3 as the target markers of Gαq-PLCβ3-mediated calcium influx, while the expression of PMCA1 and PMCA4b as the targets of calcium efflux in MDA-MB-231 cells. The results showed that the expression levels of Gαq and PLCβ3 were significantly decreased after PCI-24781 treatment (**Figure 6B-C, Figure S3F**). However, the expression levels of PMCA1 and PMCA4b were not affected by PCI-24781 (**Fig. S3E**). Of note, the activation of RGS2 was recently reported to inhibit the Gαq-PLCβ3 signaling in regulation of [Ca^2+^]_i_ 15. Consistently, our results showed the same changes in mRNA and protein expressions of RGS2 after PCI-24781 treatment, suggesting that these markers are on-target effects of the inhibition of calcium influx induced by PCI-24781 (**Figure 6B-C, Figure S3F**). To further explore the epigenetic modulation of RGS2 activation by PCI-24781, we performed quantitative chromatin immunoprecipitation (qChIP) assays with specific antibodies against H3 acetylation to examine five promoter regions of RGS2 in MDA-MB-231 and T-47D, as well as the murine breast cancer cell 4T1 cells after PCI-24781 treatment. The results showed strong binding to RGS2 #2 and #3 promoter regions of H3 acetylation after PCI-24781 treatment, indicating that PCI-24781 activates RGS2 transcription by promoting H3 acetylation binding to RGS2 promoter regions (**Figure 6D, Figure S3G**). To further investigate whether RGS2 is the target of PCI-24781, we assessed the effect of loss-of-function RGS2 with and without PCI-24781 treatment on calcium influx, carcinogenesis, and metastasis. Western blot and calcium influx assay revealed that PCI-24781 treatment slightly reversed the effect of RGS2 depletion on the protein levels of RGS2, Gαq, and PLCβ3. However, PCI-24781 treatment had no significant effect on calcium influx in RGS2-depleted cells compared with that in control cells (**Figure 6E-F**). Therefore, RGS2 depletion did not affect the amplitude of [Ca^2+^]_i_ oscillations but slightly delayed the return back to the baseline. We next examined the anti-tumor effect of loss-of-function RGS2 with and without PCI-24781 treatment by detecting apoptosis, cell proliferation, cell growth, cell invasion, and cell migration. Based on the EdU incorporation, TUNEL, colony formation, wound healing, and transwell invasion assays, we found that the inhibitory effect of PCI-24781 on carcinogenesis and metastasis in RGS2-depleted cells was much lower than that in the control cells (**Figure 6G-K**). These findings demonstrate that the anti-tumor activity and the inhibition of calcium influx by PCI-24781 are impaired in RGS2-depleted cells, indicating that RGS2 may be a potential biomarker of PCI-24781 in breast cancer cells.

### *In vivo* anti-tumor activity of PCI-24781

**Figure 7:**
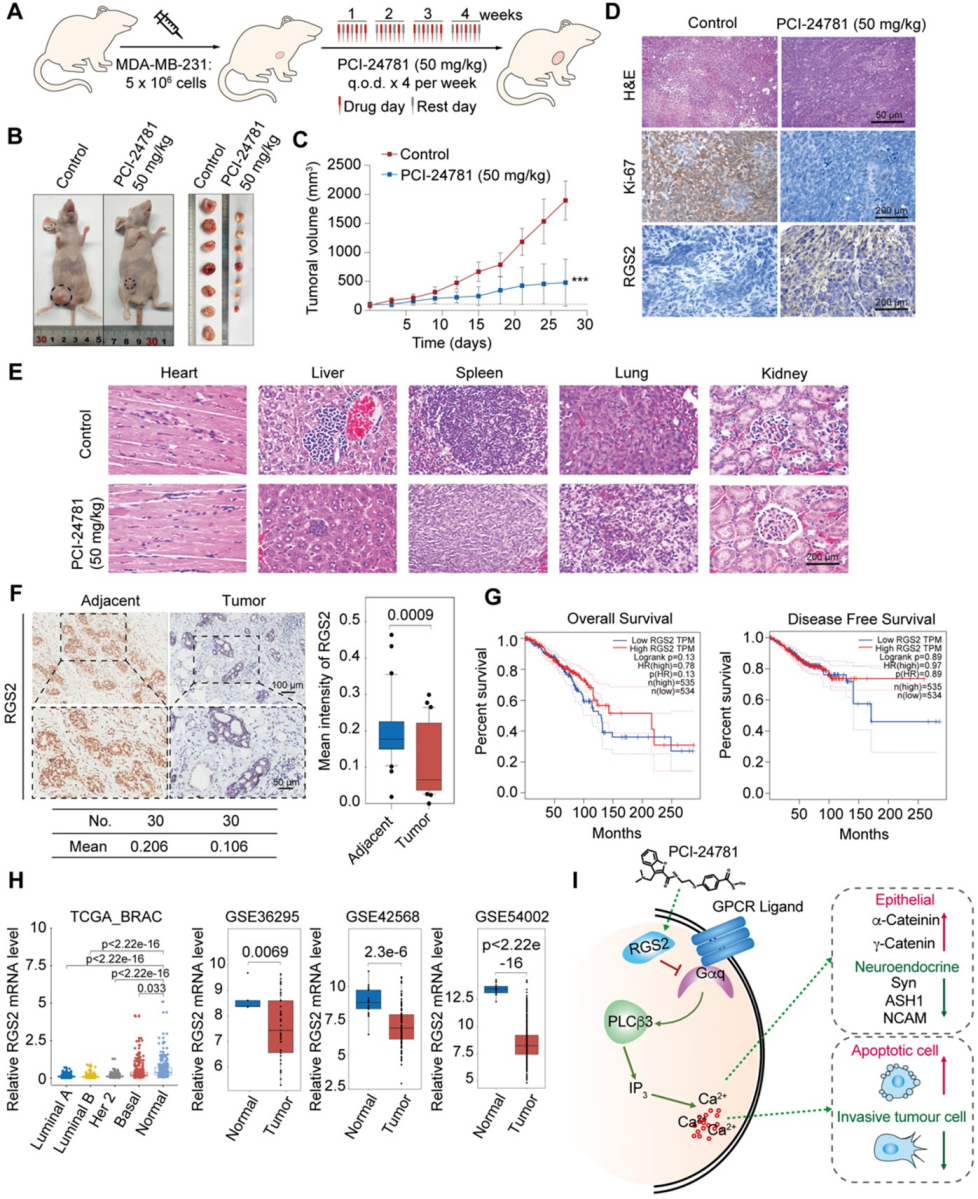
** PCI-24781 inhibits breast cancer progression *in vivo*, and RGS2 expression is reduced in breast cancer patients. A.** Experimental strategy of PCI-24781 treatment in a subcutaneous xenotransplanted tumor model. q.o.d represented injection for every other day. **B and C.** MDA-MB-231 cells were orthotopically inoculated into the abdominal mammary fat pads of 6-week-old female BALB/c nude mice (n = 5), and tumor volumes were measured weekly. When the tumor size was approximately 100 mm^3^, we initiated treatment with PCI-24781. Data are means ± SD. ***P < 0.001 at the final day. **D.** H&E staining of the tumor tissues from the control and PCI-24781 group (upper panels). Scale bars, 50 µm. IHC staining of Ki-67 and RGS2 in tumor tissues from the control and PCI-24781 group. Representative photos of two specimens are shown (lower panel). Scale bars, 200 µm. **E.** H&E staining of the heart, liver, spleen, lung, and kidney from the control and PCI-24781 group. Scale bars, 200 µm. **F.** IHC staining of RGS2 in breast carcinoma samples paired with adjacent normal mammary tissues. Representative images are shown. Scale bars, 100 µm (upper panel), 50 µm (lower panel). **G.** Overall survival and disease-free survival in the public datasets (TCGA) comparing high and low levels of RGS2 expression in breast cancer. **H.** Analysis of public datasets (TCGA BRAC, GSE36295, GS42568, and GSE54002) for the expression of RGS2 in normal and breast cancer tissues (two-tailed unpaired *t* test). **I.** Proposed mechanism by which PCI-24781 inhibits cancer by disrupting calcium influx in graphic model.

To investigate the anti-tumor effect of PCI-24781 in breast cancer *in vivo*, nude mice bearing MDA-MB-231 xenografts were injected with 50 mg/kg PCI-24781 into the caudal vein (**Figure 7A**). PCI-24781 treatment inhibited tumor growth by 74.6% compared to that in the control group (P < 0.0001) (**Figure 7B-C**), indicating a potent anti-tumor effect of PCI-24781 in breast cancer. Immunohistochemical (IHC) staining showed that RGS2 was more abundant and more intensely stained in tumor tissues from the PCI-24781-treated group than in those from the control group (**Figure 7D**). Ki-67 staining revealed that PCI-24781 administration inhibited tumor proliferation. The activation of RGS2 level and the inhibition of cellular proliferation capacity consist with* in vitro* results that PCI-24781 treatment can induce the expressions of RGS2 to disrupt the calcium signal and constrain tumor proliferation. Pathological examination showed that PCI-24781 treatment did not exhibit serious histological changes in the main organs (heart, liver, lung, spleen, and kidney (**Figure 7E**)), indicating no marked side-effects of PCI-24781 *in vivo*.

### RGS2 is downregulated in breast cancer cells

To further explore the roles of RGS2 in breast cancer tissue, we collected 30 breast cancer samples paired with adjacent tissues from patients to examine IHC assays of RGS2. The IHC results showed that the protein expression of RGS2 was downregulated in human breast cancer samples compared with that in adjacent tissues (**Figure 7F**). Kaplan-Meier survival analysis of RGS2 using the online tool GEPIA (http://gepia.cancer-pku.cn/index.html) showed that higher levels of RGS2 were correlated with increased overall survival and disease-free survival in breast cancer patients (**Figure 7G**) 17. Furthermore, lower expression levels of RGS2 in breast cancer samples compared with adjacent tissues were confirmed by four published datasets, TCGA BRAC, GSE36295, GS42568, and GSE54002 (**Figure 7H**), suggesting that RGS2 might be a candidate tumor suppressor in breast cancer. Moreover, using GEPIA, the results showed that the mRNA levels of RGS2 were also lower in many tumor types compared with corresponding adjacent tissues (**Figure S4**), indicating a potential role of RGS2 in tumor progression. In summary, these results suggest that RGS2 could be a candidate tumor suppressor in breast cancer.

## Discussion

In this study, we have shown that an epidrug PCI-24781 reduced intracellular free Ca^2+^ in breast cancer cells. Intracellular free Ca^2+^ was identified as a crucial effector of cell fate, and the modulation of calcium signaling is relevant to the development of cancer 16. Our findings suggest a mechanism through which calcium influx is modulated by epigenetic remodeling, wherein the HDAC inhibitor PCI-24781 can alter calcium signaling, thereby affecting apoptosis, cell proliferation, and cell differentiation to inhibit breast cancer progression (**Figure 7I**).

Epigenetic remodeling has become a powerful target for cancer therapy due to its reversible changes. Epidrugs or drugs that target epigenetic markers, such as HDAC inhibitors, have been proposed as a novel therapeutic approach for several malignancies, including hematological tumors 18-20. At present, the FDA has approved six HDAC inhibitors worldwide, and the main indications are T-cell lymphomas, among which panobinostat has been approved for multiple myeloma. PCI-24781 has been approved for monotherapy in the treatment of follicular lymphoma. It has been revealed that histone acetylation can stimulate transcription at the genomic loci to direct gene expression and participate in multiple cells signaling pathways for DNA damage repair and cell cycle checkpoints 21. In addition, HDAC inhibitors are potential regulators for the activity of multiple Ca^2+^ signaling, thereby inducing cell death in tumors. The HDAC inhibitor TSA increased calpain expression through histone hyperacetylation, inhibiting calpain activity, and inducing neuroblastoma cell death 22. HDAC inhibitor valproic acid (VPA) can enhance the effect of doxorubicin on autophagy of lymphoma cells by diminishing intracellular IP_3_ and blocking calcium depletion 23. However, in breast cancer, VPA exhibits diverse effects on Ca^2+^ signaling in specific subtypes. ER-negative cells do not alter their calcium pump expression and transient Ca^2+^ response upon VPA treatment. In contrast, ER-positive cells are significantly more sensitive to VPA treatment, resulting in the inhibition of calcium pumps 24. Although several HDAC inhibitors have been reported to disturb calcium signaling, the mechanisms of different inhibitors on different tumor types are still elusive. The anti-tumor mechanism of PCI-24781 as an HDAC inhibitor through Ca^2+^ signaling modification is not yet clear. We provide evidence that PCI-24781 significantly affects the intracellular calcium signal pathway in breast cancer cells. The present study on the mechanism of PCI-24781 as a calcium influx regulator might provide a drug target for improving the clinical efficacy of epidrugs against breast cancer.

[Ca^2+^]_i_ homeostasis is controlled by a variety of molecular pathways, including the Gαq-PLCβ3-mediated calcium pathway and plasma membrane-localized Ca^2+^ pumps PMCA. It has been reported that HDAC inhibitors short chain fatty acids and suberoylanilide hydroxamic acid could increase the expression of PMCA4b in MCF-7 cells and Ca^2+^ clearance via calcium efflux 25. However, our results showed that PCI-24781 mainly affected the calcium influx, indicating that different HDAC inhibitors might regulate different processes of calcium signaling. The heterotrimeric G protein comprises of α, β, and γ subunits in the inactive guanosine diphosphate-binding state. As a subfamily of G protein α subunits, Gαq activates the phospholipase activity of PLCβ3 through the interaction of GTP-bound α subunits with the C2 domain and COOH-terminal domain of PLCβ3. Subsequently, PLCβ3 catalyzes the hydrolysis of phosphatidylinositol 4,5-bisphosphate to produce the intracellular messenger IP_3_, thereby promoting the release of intracellular calcium 26. There have also been reports showing that RGS2 can reduce GPCR signaling by stimulating the GTPase activity of Gαq to further decrease PLCβ3 activity 27-29. These reports were consistent with our results, as we found that PCI-24781 treatment activated RGS2 expression, suppressed the levels of PLCβ3 and Gαq, and decreased calcium influx. Previous studies have reported that the activity of RGS2 is regulated by epigenetic modifications in prostate, testicular, and ovarian cancers 30-32. In ovarian cancer cells, HDAC inhibitors activated H3 acetylation on the RGS2 promoter to enhance the expression of RGS2 32. In addition, as a tumor suppressor in several tumors, the expression of RGS2 is low in many cancers, such as breast and prostate cancer 33. RGS2 also impedes Gq-coupled GPCR signal to inhibit the growth of androgen-independent LNCaP cells 34. The overexpression of RGS2 in breast cancer cells reduces the protein level of testicular specific Y-like protein 5 (TSPYL5) to disturb calcium pumps, thereby inhibiting cellular proliferation 35. Through bioinformatics analysis, we found that the expression of RGS2 might be related to the pathological stage of breast cancer in the breast cancer cohort of the TCGA database. The expression of RGS2 is lower in malignant breast cancer. Besides, the expression of RGS2 might be related to the survival of breast cancer patients, and the low expression of RGS2 might cause a lower survival rate and poor prognosis. Therefore, we assumed that the upregulation of RGS2 in breast cancer cells might be induced by the activation of histone acetylation after treatment with an HDAC inhibitor. Indeed, we hypothesize that epigenetic changes stimulate RGS2 expression to abate the Gαq-PLCβ3-mediated calcium pathway, which disrupts Ca^2+^ homeostasis, thus showing that epidrugs play key roles in regulating calcium signaling through epigenetic modification.

A major role of the Ca^2+^ signal is integrating with the signal-transduction cascades to affect cell fate during cancer progression, which can control cellular directional migration by regulating local calcium flickers on the edges of the cells, which, in turn, affects cell stress and sensitivity to apoptotic stimuli 11. The effects of Ca^2+^ on cell proliferation especially focus on the early G1, the G1/S, and the G2/M transition stages of the mammalian cell cycle 36, 37. During mitosis, cells are sensitive to Ca^2+^ depletion, wherein the suppression of extracellular Ca^2+^ leads to the inhibition of cell proliferation. Therefore, our results showing that epidrug treatment disrupted cell proliferation following a decrease in mitosis suggest that these effects may be induced by the depletion of Ca^2+^. As one of the key regulators of cell migration, the increase in intracellular Ca^2+^ concentration in space, time, and amplitude is considered to be linked to cell migration 38. Virtually every step of tumor cellular migration and invasion, including the rate of cellular movement and directional control, depends on a certain extent on calcium signaling 10, 39, 40. Tumor cells need to acquire the ability to promote migration by proteolysis of the ECM to acquire invasive capacity, and the most common markers found in highly invasive cancer cells are special membrane protrusions containing ECM-degrading MMPs 41, 42. Consistent with our observations, the suppression of MMP1, 2, 3, 15, and 16 might be correlated with the decrease in cellular invasion and metastasis, resulting in the weakening of cell membrane activity and inhibition of cell movement. The greatest effect of intracellular Ca^2+^ concentration changes on tumor cells is cell death, although augmented intracellular Ca^2+^ is considered to be an important condition for apoptosis 43, 44. Interference with Ca^2+^ signaling can also lead to tumor cell death. In breast cancer cell lines MCF-7 and T-47D, the interruption of Ca^2+^ transfer from the ER to the mitochondria induced cell death 13. Although [Ca^2+^]_i_ was decreased by treatment with the epidrug in our study, the inhibition of Gαq-PLCβ3-mediated calcium pathway which interrupted the release of calcium storage and Ca^2+^ transfer still amplified the sensitivity of tumor cells to apoptotic stimuli, leading to a certain degree of apoptosis. Owing to the diversity of calcium signaling in cancer progression, we speculate that the ability of PCI-24781 to inhibit the breast cancer phenotype occurs mainly through the suppression of calcium influx, which constrains cellular biological function and affects cell survival.

EMT in tumor cells refers to the transformation of epithelial cells into mesenchymal-like phenotypes, leading to enhanced motility of migration and invasion, which is the key factor for cancer metastasis. However, the transformation of tumor epithelial cells also has the characteristics of NE tumors that exhibit high malignancy and poor prognosis 45. Tumor cells that undergo NE differentiation are commonly characterized by the NE morphology and the expression of NE markers, such as NCAM1, ASH1, and Syn 46. Through the identification of NE markers, neuroendocrine breast tumors with neuroendocrine characteristics have been found in some clinical cases 47, 48. We observed that the basal lines of calcium influx in breast tumor cells and breast epithelial cells before the epidrug treatment seemed to be related to the malignancy degree of tumor cells. The calcium influx intensity in breast epithelial cells MCF-10A was the faintest, while that in MDA-MB-231 cells, which are highly malignant cells, was the strongest. Furthermore, the expression of NE differentiation markers was also detected in MDA-MB-231 cells. It has been reported that in androgen-dependent prostate cancer epithelial cells, the dysregulation of intracellular Ca^2+^ homeostasis was affected by NE differentiation, suggesting that NE differentiation could disrupt Ca^2+^ homeostasis in cancer cells 49-51. EMT is dependent on calcium signaling, and the dynamic Ca^2+^ balance is distinctly different between epithelial-like and mesenchymal-like cells. In breast cancer cells MDA-MB-468, EMT induced by EGF was linked to an increase in intracellular Ca^2+^ 52. We have observed that PCI-24781 enhanced epithelial differentiation with the upregulation of epithelial markers, while the degree of mesenchymal differentiation did not change significantly; interestingly, the landmark markers of NE differentiation were abated. To sum up, the reason why the triple negative breast cancer cell lines exhibited more sensitivity to PCI-24781 treatment, is probably because these cells have the characteristics of NE differentiation, which allows PCI-24781 to disrupt Ca^2+^ homeostasis to dysregulate calcium influx. Adjusting Ca^2+^ homeostasis by restraining [Ca^2+^]_i_ might restore the transformation of neuroendocrine differentiation into epithelial differentiation, so that cancer cells display more sensitivity to the calcium signaling feedback. The adjustment of Ca^2+^ homeostasis is a new insight mechanism of PCI-24781.

## Conclusions

Taken together, in this study, we developed an effective selection strategy for screening multiple compounds at different concentrations. By using the selection strategy, we identified an epidrug PCI-24781 through high-content analysis screening of a small molecule epigenetic inhibitor library. PCI-24781 had low toxicity to non-cancer breast epithelial cells and high efficiency against breast cancer cells *in vivo and in vitro*. We propose that the anti-tumor mechanism of PCI-24781 is based on epigenetic remodeling that results in suppression of Ca^2+^ influx and cell differentiation by activating RGS2 expression. This study provides new insight into the use of epidrugs, especially of HDAC inhibitors, for the therapy of malignancies.

## Supplementary Material

Supplementary figures and tables.

## Figures and Tables

**Table 1:** The ADME prediction of PCI-24781

Property	Predicted values	Probability	Meaning & Preference
**Absorption**			
Papp (Caco-2 Permeability)	-5.379 cm/s		Optimal: higher than -5.15 Log unit
P-gp-inhibitor	+++	0.912	The Pgp-inhibitor classification criteria refers the reference
P-gp-substrate	---	0.237	More likely to be a Pgp non-substrate
HIA (Human Intestinal Absorption)	-	0.448	≥30%: HIA+; <30%: HIA-
F (20% Bioavailability)	+	0.538	≥20%: F20+; <20%: F20-
F (30% Bioavailability)	+	0.555	≥30%: F30+; <30%: F30-
**Distribution**			
PPB (Plasma Protein Binding)	92.57%		Significant with drugs that are highly protein-bound and have a low therapeutic index.
VD (Volume Distribution)	-0.375 L/kg		<0.07L/kg: Confined to blood, Bound to plasma protein or highly hydrophilic
BBB (Blood-Brain Barrier)	++	0.868	BB ratio ≥0.1: BBB+; BB ratio <0.1: BBB-
**Metabolism**			
P450 CYP1A2 inhibitor	+	0.609	Molecules that labeled inhibitor in PubChem BioAssay were regarded as inhibitor.
P450 CYP1A2 Substrate	+	0.62	Molecules that labeled substrate in PubChem BioAssay were regarded as substrate.
P450 CYP3A4 inhibitor	---	0.218	Molecules that labeled inhibitor in PubChem BioAssay were regarded as inhibitor.
P450 CYP3A4 substrate	+	0.666	Molecules that labeled substrate in PubChem BioAssay were regarded as substrate.
P450 CYP2C9 inhibitor	+	0.514	Molecules that labeled inhibitor in PubChem BioAssay were regarded as inhibitor.
P450 CYP2C9 substrate	+	0.508	Molecules that labeled substrate in PubChem BioAssay were regarded as substrate.
P450 CYP2C19 inhibitor	+	0.549	Molecules that labeled inhibitor in PubChem BioAssay were regarded as inhibitor.
P450 CYP2C19 substrate	+	0.657	Molecules that labeled substrate in PubChem BioAssay were regarded as substrate.
P450 CYP2D6 inhibitor	+	0.556	Molecules that labeled inhibitor in PubChem BioAssay were regarded as inhibitor.
P450 CYP2D6 substrate	+	0.587	Molecules that labeled substrate in PubChem BioAssay were regarded as substrate.
**Elimination**			
T 1/2 (Half Life Time)	1.496 h		Range: <3h: low
CL (Clearance Rate)	1.285 mL/min/kg		Range: <5 mL/min/kg: low

## Abbreviations

HDACs: histone deacetylase
IC_50_: half maximal inhibitory concentration
RGS2: regulator of G-protein signaling 2
ER: estrogen receptor
MMP: matrix metalloproteinases
PLCβ: phospholipase C-β
ECM: extracellular matrix
IP_3_: inositol-1,4,5-triphosphate
DMSO: dimethyl sulfoxide
JAK: janus kinase
mTOR: mammalian target of rapamycin
HIF: hypoxia-inducible factor
TSA: trichostatin A
MEF: mouse embryonic fibroblasts
HUVEC: human umbilical vein endothelial cells
H3: histone H3
EdU: 5-ethynyl-2'-deoxyuridine
RNA-seq: RNA sequencing
GSEA: Gene Set Enrichment Analysis
EMT: epithelial-mesenchymal transition
GO: Gene Ontology
KEGG: Kyoto Encyclopedia of Genes and Genomes
NE: Neuroendocrine
GDP: guanosine diphosphate
NCAM1: neural cell adhesion protein 1
ASH1: absent small and homeotic disks protein 1 homolog
Syn: synaptophysin
PMCA: plasmalemmal Ca^2+^ pump isoform
qChIP: quantitative chromatin immunoprecipitation
VPA: valproic acid
TSPYL5: testicular specific Y-like protein 5
