# Supplementary material for: The histone deacetylase inhibitor PCI-24781 impairs calcium influx and inhibits proliferation and metastasis in breast cancer
Source: Theranostics. 2021 Jan 1;11(5):2058–76. doi: 10.7150/thno.48314 (PMC7797697; doi:10.7150/thno.48314)
Supplement: Supplementary file 1 — Supplementary figures and tables. [file thnov11p2058s1.zip › Supplementary material/Supplementary Figure.pdf]

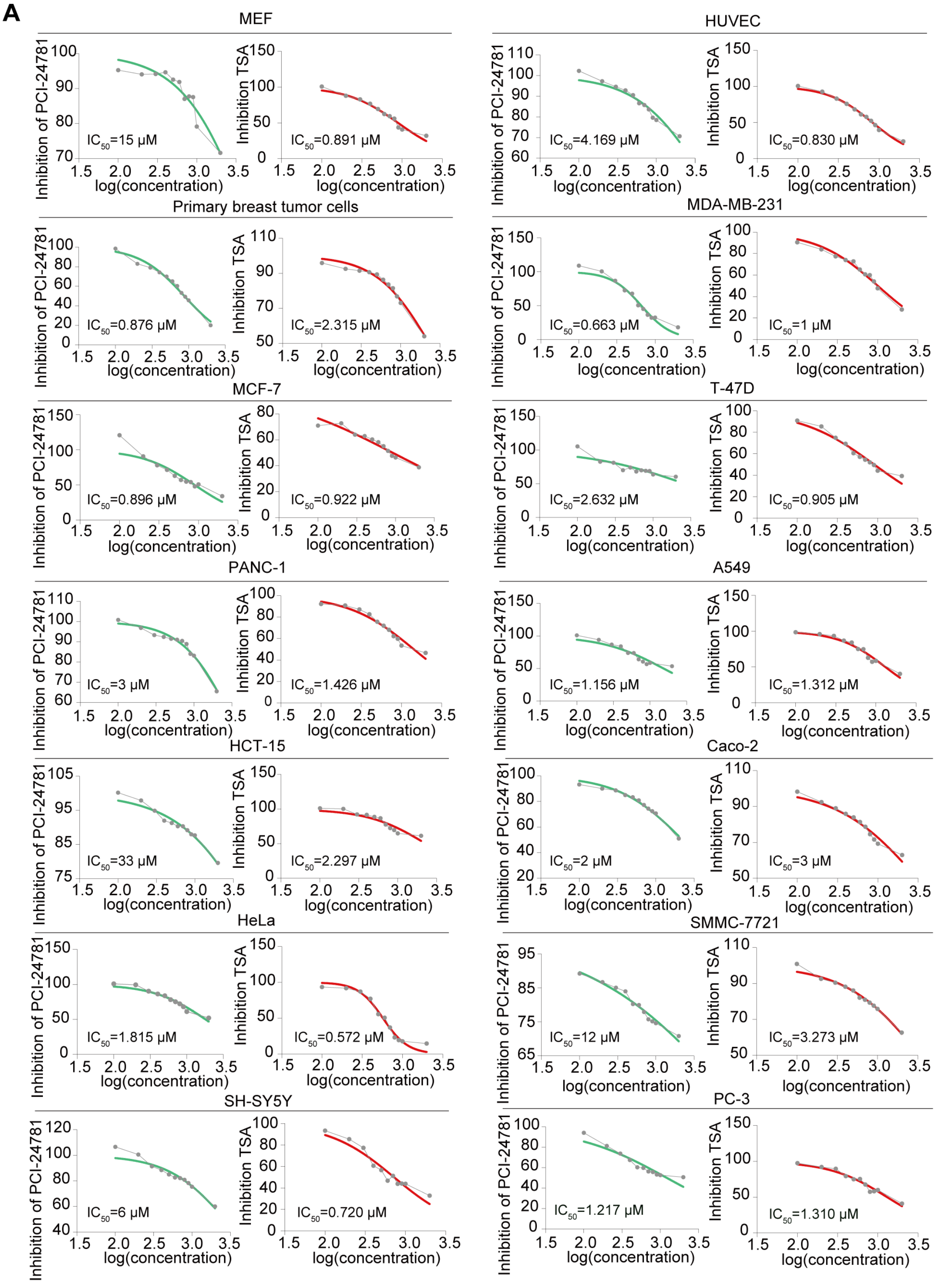

Figure S1. PCI-24781 is selective to breast cancer cells. A. Measurement of IC_{50} by CCK8 assay in cell panels (including MEF, HUVEC, primary breast tumor cells, MDA-MB-231, MCF-7, T-47D, PANC-1, A549, HCT-15, Caco-2, HeLa, SMMC-7721, SH-SY5Y, and PC-3) treated with PCI-24781 and TSA (n = 3).

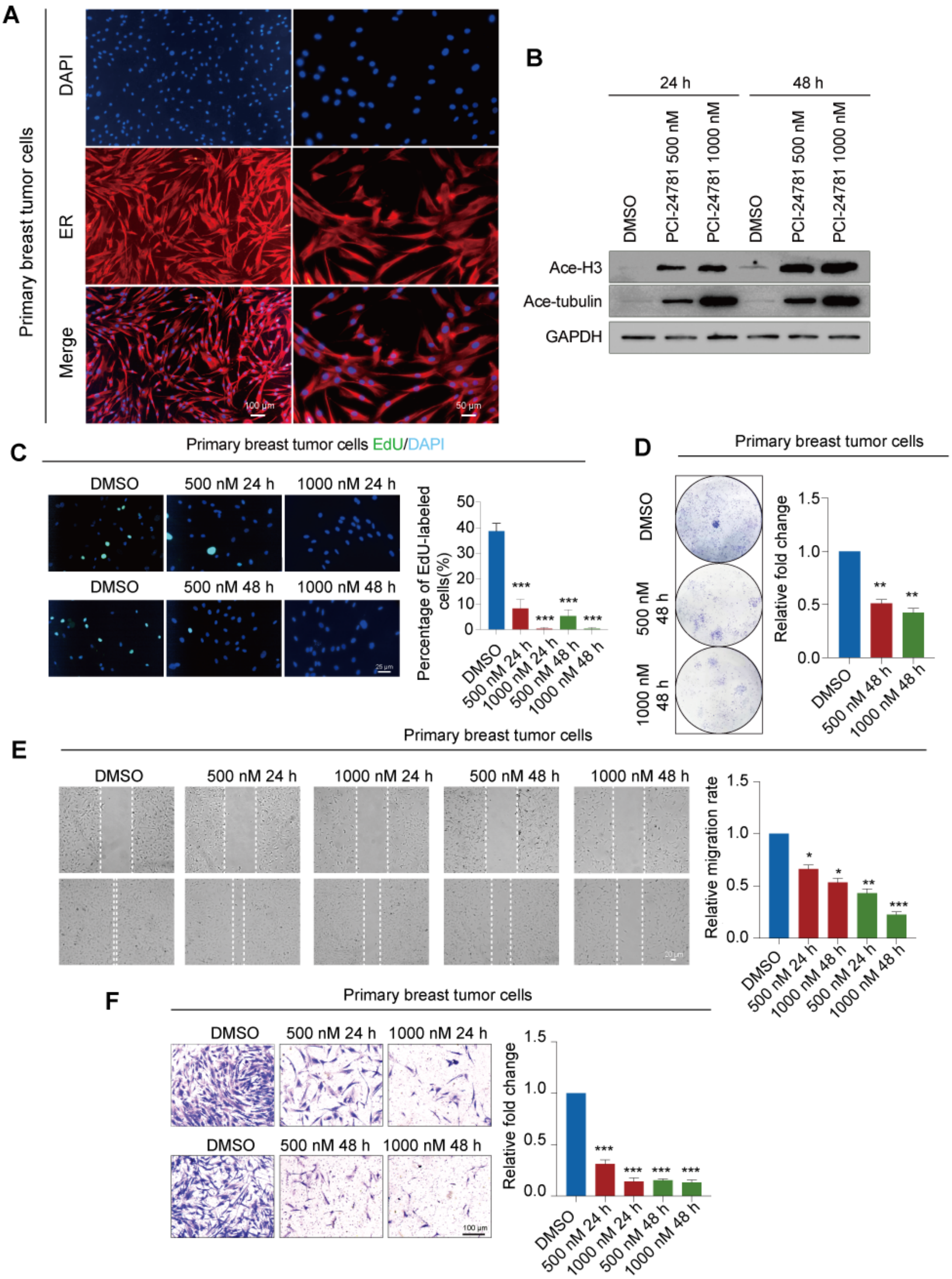

Figure S2. PCI-24781 treatment inhibits breast carcinogenesis and metastasis in primary breast tumor cells. A. Immunofluorescence assay was carried out with anti-ER antibody in primary breast tumor cells. Scale bars, 100 μm (left images), 50 μm (right images). B. Western blot using the indicated antibodies were performed on total protein extracted from primary breast tumors cells treated with PCI-24781. Ace-H3, acetylated histone H3; Ace-α-tublin, acetylated α-tublin. C. EdU incorporation assays were performed on primary breast tumor cells treated with PCI-24781. Representative images are shown on the left, and statistical analysis is shown on the right (***P<0.001). Scale bars, 25 μm. D. Primary breast tumor cells treated with PCI-24781 were

cultured for 13 days prior to crystal violet staining. Representative images are shown on the left, and statistical analysis is shown on the right (**P<0.01). E. Wound-healing assays were performed in primary breast tumor cells treated with PCI-24781. Representative images are shown on the left, and statistical analysis is shown on the right (*P<0.05, **P<0.01, ***P<0.001). Scale bars, 20 μm. F. Cell invasion assays were performed using the matrigel transwell filters in primary breast tumor cells treated with PCI-24781. Invading cells were stained and counted. Representative images are shown on the left, and statistical analysis is shown on the right (***P<0.001). Scale bars, 100 μm.

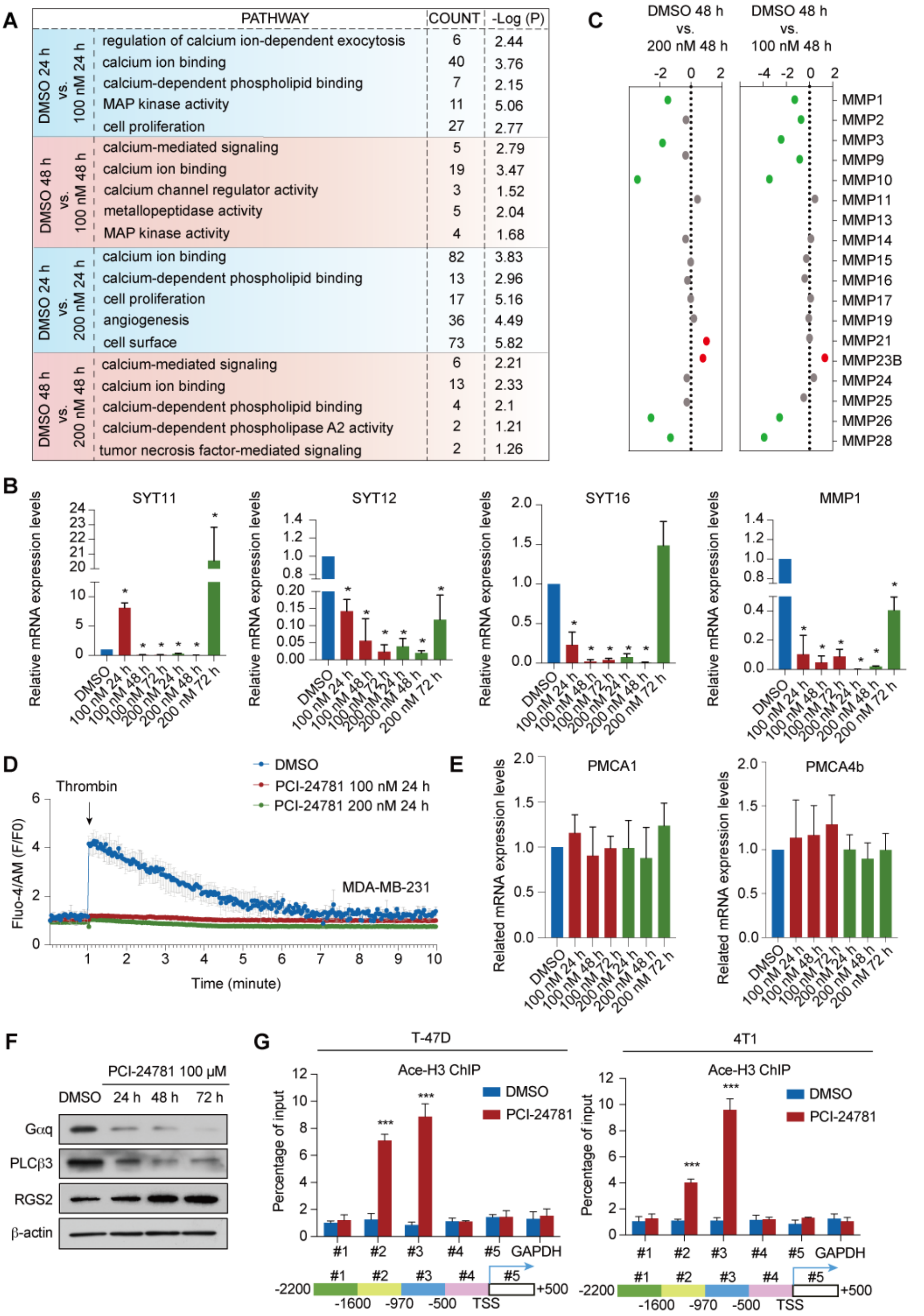

Figure S3. Genome-wide identification of transcription signaling for PCI-24781 treatment on MDA-MB-231 cells. A. List of the top 5 pathways from GO and KEGG pathway analysis. B. RT-qPCR analyses for the expression of downregulated transcription targets of PCI-24781 in MDA-MB-231 cells. Bars represent the mean ± SD of triplicate cell cultures (*P < 0.05). C. Analysis of the expression of MMP families in RNA-seq analysis. D. The frequency and amplitude of [Ca2+]i oscillations in response to thrombin stimulation in MDA-MB-231 with PCI-24781 treatment. E. RT-qPCR analyses of PMCA1 and PMCA4b expression in MDA-MB-231 cells treated with PCI-24781. Bars represent the mean ± SD of triplicate cell cultures (*P < 0.05). F. Western blot with indicated antibodies of total protein extracted from MDA-MB-231 cells treated with 100 μM PCI-24781. G. qChIP-based promoter-walk assays in T-47D and 4T1 cells after PCI-24781 treatment to map Ace-

H3 enrichment in regions #2 and #3 of the RGS2 promoter. Error bars represent the mean ± SD of three independent experiments (***P < 0.001).

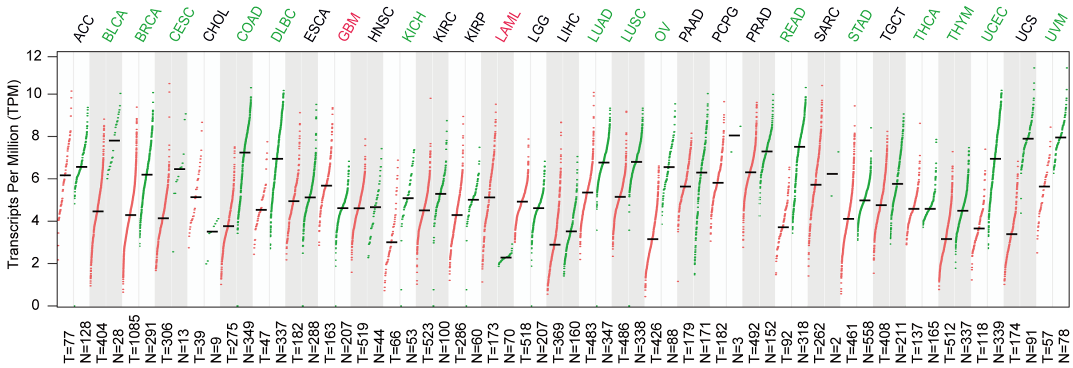

Figure S4. The RGS2 expression profile across all tumor samples. The gene expression profile across all tumor samples and paired normal tissues from GEPIA database (http://gepia.cancer-pku.cn/detail.php?gene=RGS2). Label green represent low expression compared with normal tissues. Label red represent high expression compared with normal tissues.
